# Identifying the mediating role of immune cells on the relationship between plasma lipidomes and PCOS: a two-step Mendelian randomization analysis

**DOI:** 10.1186/s13048-025-01884-z

**Published:** 2025-12-11

**Authors:** Lidan Liu, Bo Liu, Mujun Li, Lang Qin

**Affiliations:** Guangxi Reproductive Medical Center, The First Affiliated Hospital of Guangxi Medical University, Nanning, China

**Keywords:** Polycystic ovary syndrome (PCOS), Lipidome, Immune phenotype, Mendelian randomization

## Abstract

**Purpose:**

Polycystic Ovary Syndrome (PCOS) is a common endocrine disorder, with dysregulated lipid metabolism and immune dysfunction. However, it remains unclear whether immune phenotypes mediate the relationship between lipidomes and PCOS.

**Methods:**

A two-step Mendelian Randomization analysis was employed to explore the causal relationship between plasma lipidomes and PCOS and to investigate the mediating role of immune cells. A total of 179 plasma lipidomes and 731 immune phenotypes were analyzed. We used single nucleotide polymorphisms (SNPs) associated with plasma lipidome levels as instrumental variables and applied statistical methods, including the inverse-variance weighted approach, to assess potential causal relationships.The function of immune phenotypes in regulating the relationship between lipids and PCOS was evaluated through mediation analysis.

**Results:**

Ten lipid-immune pathways mediating the association between plasma lipidomes and PCOS were identified. Elevated levels of phosphatidylcholines and triacylglycerols increased the risk of PCOS by modulating immune markers such as HLA DR on B cells and CD28 on regulatory T cells. Conversely, phosphatidylinositol (18:1_18:2) demonstrated a protective effect against PCOS through CD33 on myeloid-derived suppressor cells. Six specific plasma lipidomes were causally linked to PCOS risk, including phosphatidylcholine (18:1_20:4) and triacylglycerol (50:4), which increased risk, and phosphatidylinositol (18:1_18:2), which lowered risk. Additionally, 31 immune phenotypes were identified as causally associated with PCOS, with 27 increasing risk and 4 offering protective effects.

**Conclusion:**

This study provides evidence that immune phenotypes mediate the relationship between plasma lipidomes and PCOS. These findings highlight the potential of targeting both lipid metabolic processes and immune pathways as novel therapeutic strategies for managing PCOS.

**Supplementary Information:**

The online version contains supplementary material available at 10.1186/s13048-025-01884-z.

## Introduction

 Polycystic Ovary Syndrome (PCOS) is a prevalent endocrine disorder in women of reproductive age, with a worldwide prevalence of 12–13% [24]. PCOS includes a heterogeneous collectivity of conditions that vary from hyperandrogenism to irregular menses and polycystic ovaries, and ultimately leading to infertility and metabolic dysfunction [27]. The etiology of PCOS is still unknown, but overall it is considered a multifactorial disorder caused by the intricate interplay between genetic, hormonal, and environmental factors [22]. Besides reproductive dysfunction, PCOS patients are susceptible to developing insulin resistance, dyslipidemia, and an increased risk of type 2 diabetes and cardiovascular disease [22]. The metabolic dysfunction indicates the systemic effects of PCOS, demonstrating that its influence reaches well outside the realm of reproductive well-being.

The heterogeneity of PCOS symptoms makes diagnosis and treatment challenging, highlighting the necessity of further research into the syndrome’s underlying processes. Among these, the dysregulation of lipid metabolism has emerged as a critical factor, particularly in the metabolic aspects of the disorder [10]. Women with PCOS commonly exhibit abnormal lipid profiles, including elevated triglycerides, increased low-density lipoprotein (LDL), and reduced high-density lipoprotein (HDL) [12]. These lipid abnormalities are closely linked to insulin resistance, which in turn exacerbates hyperandrogenism and disrupts normal ovarian function [1]. Recent studies suggest that altered lipidomic profiles contribute to an inflammatory and oxidative stress environment, which negatively affects ovarian steroidogenesis and follicular development [18]. Additionally, dysregulated lipid metabolism in adipose tissue leads to adipocyte hypertrophy and increases the secretion of pro-inflammatory cytokines, further aggravating insulin resistance [8]. Although these findings suggest a strong link between lipid metabolism and PCOS, the direct relationship between lipidome levels and PCOS risk remains inconclusive, necessitating further research into lipidomics as a potential biomarker for the syndrome.

In addition to lipid metabolism, immune dysregulation plays a crucial role in the progression of PCOS [11]. Chronic low-grade inflammation, a key feature of the syndrome, is driven by immune cell abnormalities that contribute to both its reproductive and metabolic characteristics [26]. Elevated levels of pro-inflammatory immune cells, such as macrophages and Th1 cells, have been found in the ovarian and adipose tissues of women with PCOS [13]. These immune cells release inflammatory cytokines, such as tumor necrosis factor-alpha (TNF-α), interleukin-6 (IL-6), and interleukin-1β (IL-1β), which impair insulin signaling and exacerbate androgen excess [5, 17]. Furthermore, immune cells directly interact with ovarian cells, driving excessive androgen production—a central feature of PCOS [3]. This immune-driven inflammation creates a vicious cycle, where metabolic dysfunction worsens the inflammatory response, accelerating the progression of PCOS.

This study utilizes a two-step Mendelian Randomization (MR) approach to investigate the causal relationship between lipidome levels and PCOS risk, as well as the mediating role of immune cells in this interaction. Mendelian Randomization is a genetic epidemiological technique that employs genetic variants as instrumental variables (IVs) to infer causality between exposures (lipidome levels) and outcomes (PCOS), while reducing the potential for confounding factors. By exploring the interplay between lipid metabolism, immune responses, and PCOS pathogenesis, this research aims to support the development of targeted therapeutic strategies for this complex disorder.

## Methods

### Study design

**Fig. 1:**
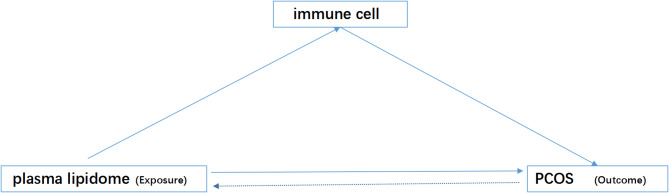
A flowchart illustrating the mediation analysis of immune phenotypes in the relationship between plasma lipidomes and PCOS

This study employed a two-sample Mendelian Randomization (MR) analysis to investigate the causal relationships between 179 plasma lipidomes and 731 immune cell phenotypes in the context of PCOS (Fig. 1). Single nucleotide polymorphisms (SNPs) significantly associated with the exposures were selected as instrumental variables (IVs). To ensure the robustness of the analysis, SNPs in linkage disequilibrium (LD) or classified as weak instruments were excluded. For reliable causal inference in MR, three key assumptions must be met: (1) the IV must be strongly associated with the exposure, (2) it should be independent of confounding factors, and (3) it must influence the outcome only through the exposure without any direct effect on the outcome. Additionally, we performed mediation MR analyses to explore the causal pathways between specific plasma lipidomes and PCOS, mediated by immune cell activity. Our findings were reported according to the STROBE-MR guidelines [23]. As the original studies received ethical approval and this analysis used publicly available data, no additional ethical approval was required.

## GWAS summary data sources

### Exposure source

Plasma lipidomics data were analyzed using both univariate and multivariate genome-wide association studies (GWAS) of 179 lipid species across 13 lipid classes from a cohort of 7,174 Finnish individuals enrolled in the GeneRISK project. Genome-wide association analysis of plasma lipidome identifies 495 genetic associations [20].

### Outcome source

GWAS summary data for polycystic ovary syndrome (PCOS) were obtained from the FinnGen study (R11 release) (https://finngen.gitbook.io/). The dataset includes genomic data from 254,618 Finnish individuals, comprising 38,322 infertility cases and 216,296 controls, covering 21,300,352 genetic variants [15].

### Mediator source

The mediator consisted of 731 immune cell phenotypes, with summary statistics retrieved from the public GWAS catalog (accession numbers GCST0001391 to GCST0002121). These immune phenotypes are categorized into seven groups: B cells, conventional dendritic cells (cDCs), various maturation stages of T cells, monocytes, myeloid cells, TBNK (T cells, B cells, natural killer cells), and regulatory T cells (Tregs) [19].

### Instrumental variable selection

A rigorous selection process was employed to ensure the validity of SNPs as instrumental variables (IVs) for the MR analysis, strictly adhering to the core MR assumptions. SNPs strongly associated with the exposure variables were identified using the extract_instruments function, applying a relaxed significance threshold of *P* < 1 × 10^-^⁵ [6]. His less stringent threshold was used to increase the number of effective IVs, considering the limited availability of SNPs at the conventional threshold of *P* < 5 × 10⁻⁸. The relaxed threshold allowed for a larger sample size, thereby enhancing the power of the analysis.

To ensure the independence of IVs and reduce confounding from linkage disequilibrium (LD), an LD analysis was conducted with a stringent threshold of r² < 0.001. A clumping procedure was implemented over a 10,000 kb window, retaining only the SNP with the lowest *P*-value in each LD block. This strategy minimizes the risk of bias from correlated SNPs.

During the alignment of SNPs between exposure and outcome datasets, SNPs with palindromic sequences or significant allele frequency discrepancies were excluded to avoid misalignment issues. To further assess the robustness of the selected IVs, F-statistics and variance explained (R²) were calculated for each SNP. This step ensured that the chosen instruments were sufficiently strong, reducing the risk of bias from weak instruments [21].

$$\:\mathrm{F}\:=\:\frac{{\mathrm{R}}^{2}\times\:(\mathrm{N}\:-\:2)}{1\:-\:{\mathrm{R}}^{2}}$$

$$\:{\mathrm{R}}^{2}=\frac{2\:\cdot\:{{\upbeta\:}}^{2}\cdot\:\:\mathrm{E}\mathrm{A}\mathrm{F}\:\cdot\:(1\:-\:\mathrm{E}\mathrm{A}\mathrm{F})}{2\:\cdot\:\mathrm{N}\:\cdot\:\mathrm{E}\mathrm{A}\mathrm{F}\:\cdot\:(1\:-\:\mathrm{E}\mathrm{A}\mathrm{F})\cdot\:{\mathrm{S}\mathrm{E}}^{2}}\:$$

### Statistical analysis

Bidirectional causal relationships between plasma lipidomes and PCOS were explored using the TwoSampleMR package (version 0.6.6) in R (version 4.4.1). Several MR methods were employed, including MR-Egger, weighted median, inverse-variance weighted (IVW), simple mode, and weighted mode. The IVW method was the primary analytical approach [9], and a causal relationship was considered significant when the IVW results showed *P* < 0.05. Additionally, the Bayesian Weighted Mendelian Randomization (BWMR) method was applied to validate the findings.

A two-step MR approach was used for mediation analysis. First, we estimated the causal effect of immune cells on PCOS (β2), followed by the causal effect of plasma lipidomes on immune cells (β1). The delta method was applied to evaluate the significance of the mediation effect (β1 × β2) and to determine the proportion of the mediation effect relative to the total effect [7].

Cochran’s Q statistic was used to assess heterogeneity among instrumental variables, with *P* < 0.05 indicating significant heterogeneity [14]. Horizontal pleiotropy was assessed using the MR-Egger intercept, where a *P*-value >0.05 suggested no evidence of pleiotropy [4]. The MR-PRESSO method was employed to detect and correct for outliers. A *P*-value greater than 0.05 indicated no significant outliers, and these data points were included in further analysis.A leave-one-out sensitivity analysis was performed, systematically removing each SNP to assess its influence on the overall results [28]. Finally, scatter plots were generated to demonstrate that results were not driven by outliers, and funnel plots were used to evaluate the robustness of associations and the absence of heterogeneity [16].

## Results

Five distinct Mendelian randomization (MR) methods—Inverse-Variance Weighted (IVW), MR-Egger regression, Weighted Median, Weighted Mode, and Simple Mode—were employed using rigorous instrumental variable selection criteria to evaluate the mediating role of 731 immune cell types in the association between 179 plasma lipidomes and polycystic ovary syndrome (PCOS).

### Effects of plasma lipidomes on PCOS

**Fig. 2:**
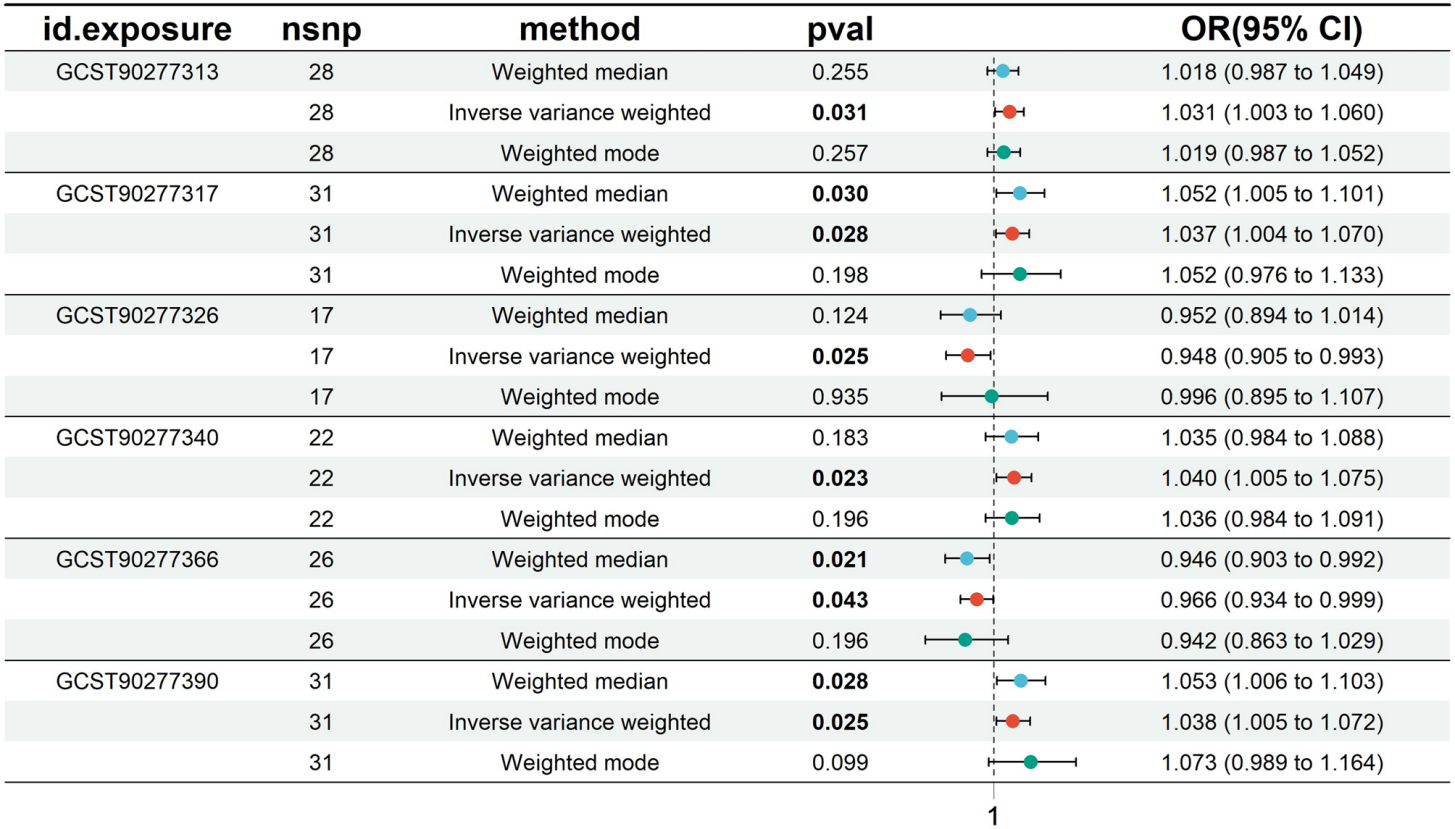
Forest plot depicting the causal effects of plasma lipidomes on the risk of PCOS

Based on IVW results (*p* < 0.05) and consistent effect direction across all five MR methods, along with no evidence of pleiotropy (*p* > 0.05) or outliers from MR-PRESSO analysis (*p* > 0.05), six plasma lipidomes were identified. Two lipidomes, Phosphatidylcholine (O-16:1_18:0) (b = -0.053, OR = 0.948, 95% CI = 0.905–0.993, *p* = 0.025) and Phosphatidylinositol (18:1_18:2) (b = -0.035, OR = 0.966, 95% CI = 0.934–0.999, *p* = 0.043), were negatively associated with PCOS risk. In contrast, four lipidomes—Phosphatidylcholine (18:1_20:4) (b = 0.030, OR = 1.031, 95% CI = 1.003–1.060, *p* = 0.031), Phosphatidylcholine (18:2_20:4) (b = 0.036, OR = 1.037, 95% CI = 1.004–1.070, *p* = 0.028), Triacylglycerol (50:4) (b = 0.037, OR = 1.038, 95% CI = 1.005–1.072, *p* = 0.025), and Phosphatidylcholine (O-18:1_20:4) (b = 0.039, OR = 1.040, 95% CI = 1.005–1.075, *p* = 0.023)—were positively associated with increased PCOS risk (Fig. 2). Subsequent Bayesian Weighted Mendelian Randomization (BWMR) analysis showed *p*-values for all six lipidomes to be less than 0.06, warranting further investigation (Supplementary Table 1). Forest plots, scatter plots, leave-one-out analysis, and funnel plots further validated these findings (Figure S1,Figure S2,Figure S3, Figure S4).

### Effects of PCOS on plasma lipidomes

No reverse causal relationship was identified between the six plasma lipidomes and PCOS (*p* > 0.05).

### Effects of immune phenotypes on PCOS

**Fig. 3:**
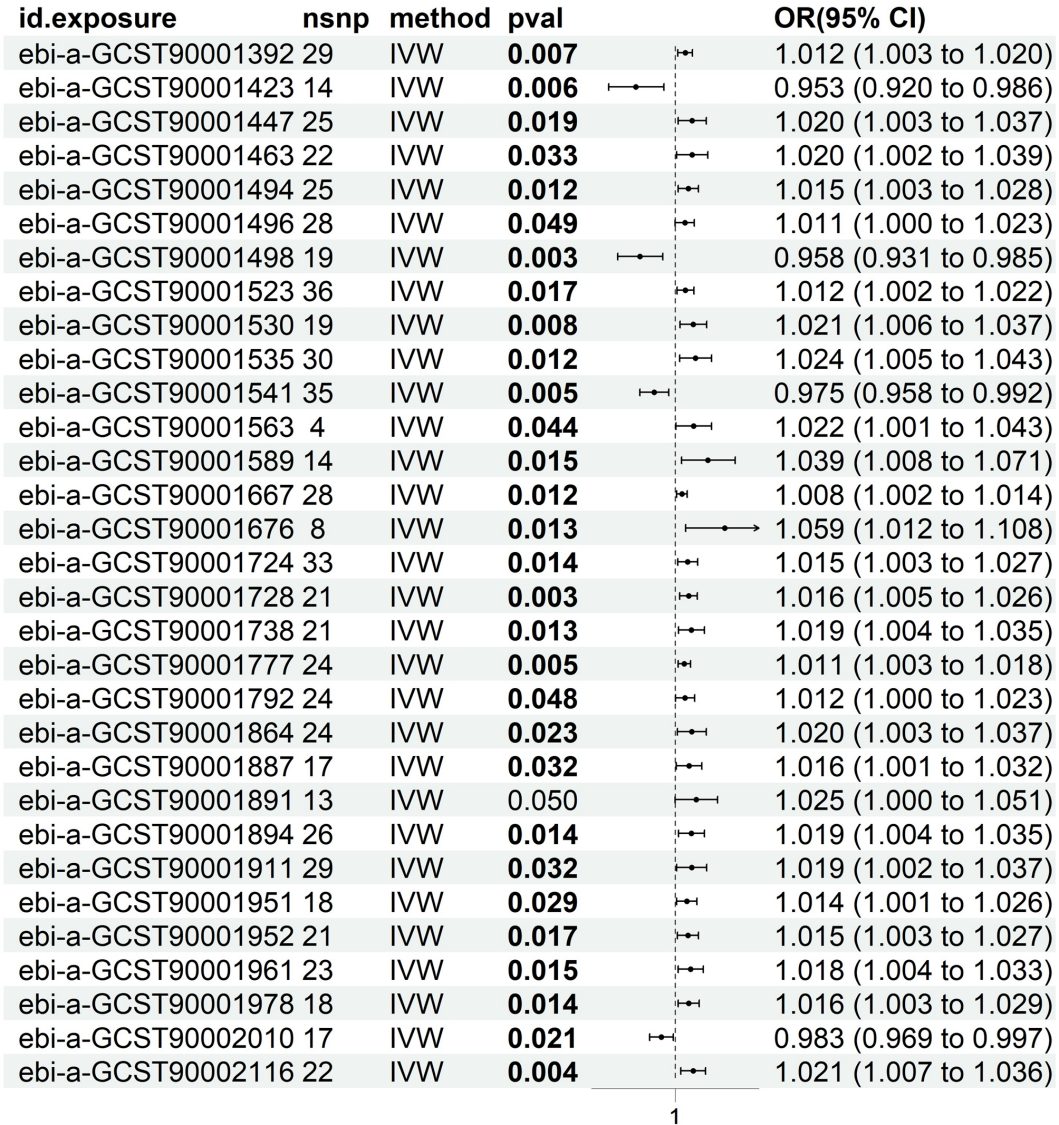
Forest plot depicting the causal effects of immune phenotypes on the risk of PCOS

Based on IVW results (*p* < 0.05) and consistent effect direction across all five MR methods, with no evidence of pleiotropy (*p* > 0.05) or outliers from MR-PRESSO analysis (*p* > 0.05), 31 immune phenotypes were identified. Of these, 27 immune phenotypes were positively associated with increased PCOS risk, while four were negatively associated with PCOS (Fig. 3).

### Effects of plasma lipidomes on immune phenotypes

**Fig. 4:**
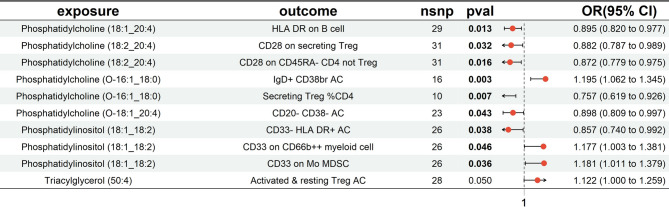
Forest plot depicting the causal effects of plasma lipidomes on immune phenotypes

We conducted further analysis using plasma lipidomes identified as associated with PCOS and examined their relationship with PCOS-related immune phenotypes. A two-sample MR analysis was performed, using plasma lipidomes as the exposure and immune phenotypes as the outcome. Based on IVW results (*p* < 0.05) and consistent effect direction across all five MR methods, along with tests for pleiotropy (*p* > 0.05) and MR-PRESSO results (*p* > 0.05), ten plasma lipidome-immune phenotype relationships were identified (Fig. 4).

### The mediation effect of immune phenotypes in the causal association between plasma lipidomes and PCOS

A two-step MR approach was employed for mediation analysis to investigate whether the causal relationship between plasma lipidomics and PCOS is mediated by specific immune phenotypes. The analysis identified 10 mediation pathways where immune phenotypes play a critical role in modulating the impact of lipid species on PCOS development (Table 1).

**Table 1:** The mediation effect of immune phenotype in the causal association between the plasma lipidomes and PCOS

Exposure	Mediator	Outcome	Total effect(beta_all)	Mediation effect	
				Beta1	Beta2
Phosphatidylcholine (18:1_20:4)	HLA DR on B cell	PCOS	0.304	-0.111	0.021
Phosphatidylcholine (18:2_20:4)	CD28 on secreting Treg	PCOS	0.036	-0.125	0.016
Phosphatidylcholine (18:2_20:4)	CD28 on CD45RA- CD4 not Treg	PCOS	0.036	-0.137	0.025
Phosphatidylcholine (O-16:1_18:0)	IgD + CD38br AC	PCOS	-0.053	0.178	0.012
Phosphatidylcholine (O-16:1_18:0)	Secreting Treg %CD4	PCOS	-0.053	-0.279	0.015
Phosphatidylcholine (O-18:1_20:4)	CD20- CD38- AC	PCOS	0.039	-0.108	-0.048
Phosphatidylinositol (18:1_18:2)	CD33- HLA DR + AC	PCOS	-0.035	-0.155	0.012
Phosphatidylinositol (18:1_18:2)	CD33 on CD66b + + myeloid cell	PCOS	-0.035	0.163	0.013
Phosphatidylinositol (18:1_18:2)	CD33 on Mo MDSC	PCOS	-0.035	0.166	0.015
Triacylglycerol (50:4)	Activated & resting Treg AC	PCOS	0.037	0.115	-0.043

Beta_all: Effects of exposure on outcome

Beta1: effects of exposure on mediator

Beta2: effects of mediator on outcome

Each mediation pathway includes three components: plasma lipidome (exposure), immune phenotype (mediator), and PCOS (outcome). The lipidomes involved include phosphatidylcholines, phosphatidylinositols, and triacylglycerols, while the immune phenotypes encompass markers such as HLA DR on B cells, CD28 on regulatory T cells (Tregs), and CD33 on myeloid-derived suppressor cells (MDSCs). The Beta1 and Beta2 values quantify how these lipidomes indirectly influence PCOS development through immune modulation.

Certain plasma lipidomes were found to increase PCOS risk via their effects on specific immune phenotypes. For instance, Phosphatidylcholine (18:1_20:4) increases PCOS risk by modulating HLA DR expression on B cells. Similarly, Triacylglycerol (50:4) is positively associated with PCOS risk through its influence on both activated and resting Tregs. In contrast, other pathways suggest that specific lipids may exert protective effects against PCOS via immune regulation. Notably, Phosphatidylinositol (18:1_18:2), by modulating CD33 on MDSCs, is negatively correlated with PCOS risk, indicating its potential role in reducing disease risk.

## Discussion

The research offers new insights about the interrelationship of plasma lipidomes, immune phenotypes, and PCOS development using a two-step Mendelian Randomization (MR) strategy. The evidence supports that certain immune cells are the mediators between plasma lipidomes and PCOS and that 10 mediation pathways are involved. The findings shed new light on the pathophysiological processes involved in PCOS, especially on how immune modulation might play a bridge between lipid metabolism and reproductive dysfunction.

### Plasma lipidomes and PCOS

Previous studies have proven a strong correlation between dysregulated lipid metabolism and the metabolic characteristics of PCOS, especially its relationship to hyperandrogenism and insulin resistance [10]. We, however, affirmed the causal role of several lipidomes, such as phosphatidylcholines and triacylglycerols, in increasing PCOS risk. Of specific interest was the finding that phosphatidylcholine (18:1_20:4) and triacylglycerol (50:4) would have a positive effect on PCOS risk, which is consistent with previous evidence that a disrupted lipid profile is responsible for both endocrine and metabolic features of the syndrome [10]. These lipids can affect the activity of ovaries by impairing follicle development and steroidogenesis and enhancing hyperandrogenism and metabolic abnormalities, which are typical attributes of PCOS [2].

Interestingly, some lipid species displayed protective activity against PCOS. For example, phosphatidylinositol (18:1_18:2) was inversely correlated with risk for PCOS via its actions on immune cells, such as myeloid-derived suppressor cells (MDSCs). This results highlights the possible role of individual lipid molecules against the pro-inflammatory condition commonly linked to PCOS. Additional study is indicated to investigate how these protective lipid species help to reverse metabolic and hormonal balance in PCOS patients.

While this research supports that dysregulated lipid metabolism is involved in the pathophysiology of PCOS, it is also important to consider the heterogeneity of PCOS phenotypes, including lean PCOS.The lean PCOS phenotypes are not characterized by obesity or evident insulin resistance but can exhibit hormonal and metabolic abnormalities, such as disrupted lipid profiles [25]. There is evidence that lean PCOS patients may have inherent abnormalities within lipid metabolism at the cellular or molecular level, regardless of adiposity. Our findings based on genetic instruments free from BMI indicate that specific lipidomic changes may affect the risk for PCOS regardless of systemic insulin resistance or obesity. Therefore, the causal associations between specific lipid species and PCOS may also be pertinent to lean PCOS subtypes, affirming the use of lipidomic markers throughout the entire clinical spectrum.

### Immune phenotypes and PCOS

The role of chronic low-grade inflammation in PCOS has been increasingly recognized, with immune dysregulation contributing to both reproductive and metabolic dysfunction [11]. This study builds on that knowledge by identifying specific immune phenotypes that mediate the relationship between lipid species and PCOS. Among the immune phenotypes identified, HLA DR on B cells and CD28 on regulatory T cells (Tregs) were particularly notable in modulating the effect of lipids like phosphatidylcholines on PCOS risk. These findings align with earlier studies that found elevated levels of activated immune cells in PCOS patients, which secrete pro-inflammatory cytokines that impair insulin signaling and promote androgen excess [29].

The identification of MDSCs as mediators in a protective lipid pathway highlights the dual role of immune cells in PCOS pathogenesis. MDSCs are known to suppress excessive immune activation and maintain immune homeostasis [19]. In this study, CD33 on MDSCs mediated the protective effect of phosphatidylinositol (18:1_18:2) on PCOS, suggesting that enhancing the activity of these cells could mitigate the inflammatory environment typically seen in PCOS.

### Implications for PCOS management

The implications of this research are clinically significant. By identifying the specific immune phenotypes and lipid species implicated in PCOS risk, new therapeutic opportunities for personalized medicine are available. Lipidomics would be used as a potential biomarkeror tool for risk stratification for PCOS and immune modulatory therapies that target specific inflammatory processes involved in PCOS may be developed. For example, immunotherapies aimed at reducing B cell activation or that augment suppressive function of Tregs and MDSCs may represent new therapeutic strategies to correct both metabolic and reproductive function abnormalities associated with PCOS.

Furthermore, the use of Mendelian Randomization provides definitive evidence of causality, reducing the possibility that these associations are confounded associations. Though being statistically significant, However, further clinical and experimental evidence is necessary for the translation of these observations to therapeutic application. It is also worth mentioning that the study used predominantly European-population controls, which may limit the application of the findings to other cohorts.

### Strengths and limitations

The principal strength of the study is its application of a two-step Mendelian Randomization design,, which enabled us to probe the causal relationships between PCOS, immune phenotypes, and plasma lipidomes. The application of GWAS data on large and well-characterized cohorts adds to the credibility of the findings. The use of multiple methods for MR,, such as IVW and MR-Egger, also ensured the quality of the findings by avoiding biases due to pleiotropy and heterogeneity.

However, several limitations need to be kept in mind. Firstly, while being a very useful tool for assessing causality, MR relies on the assumption that genetic variants employed as instrumental variables act on the outcome only via exposure of interest. While we used various approaches to check for pleiotropy, it still cannot be excluded. Secondly, the study is constrained to a specific population subgroup, and thus the generalizability to various ethnic groups may be limited. The findings need to be replicated in more diverse groups in follow-up studies. Thirdly, the use of summary-level data excludes the analysis of interactions between various lipid species or immune phenotypes, which would give us a better insight into PCOS pathogenesis. Finally, the potential for reverse causation cannot be fully excluded. Chronic low-grade inflammation in PCOS may alter lipid metabolism through immune-mediated mechanisms. While our analysis focused on the lipidome-to-immune-to-PCOS direction, future bidirectional MR studies are needed to clarify the potential feedback effects of immune dysregulation on lipidomic profiles.

## Conclusion

In summary, this study identifies novel lipid-immune pathways that mediate the causal relationship between plasma lipidomes and PCOS, providing new insights into the biological mechanisms underlying this complex disorder. The findings underscore the potential of targeting both lipid metabolism and immune dysregulation in the development of therapeutic strategies for PCOS. Further experimental studies and clinical trials are needed to validate these results and explore the therapeutic potential of lipid-immune modulation in the management of PCOS.

## Supplementary Information

Additional file 1: Figure S1. Forest plot.

Additional file 2: Figure S2. Scatter plot.

Additional file 3: Figure S3. Sensitivity_analysis plot.

Additional file 4: Figure S4. Funne lplo.

Additional file 5: Supplement table 1. BWMR results of plasma lipidomes on PCOS.

## Data Availability

No datasets were generated or analysed during the current study.
