# Supplementary figures and images for: Identifying the mediating role of immune cells on the relationship between plasma lipidomes and PCOS: a two-step Mendelian randomization analysis
Source: J Ovarian Res. 2025 Dec 11;19:16. doi: 10.1186/s13048-025-01884-z (PMC12817645; doi:10.1186/s13048-025-01884-z)

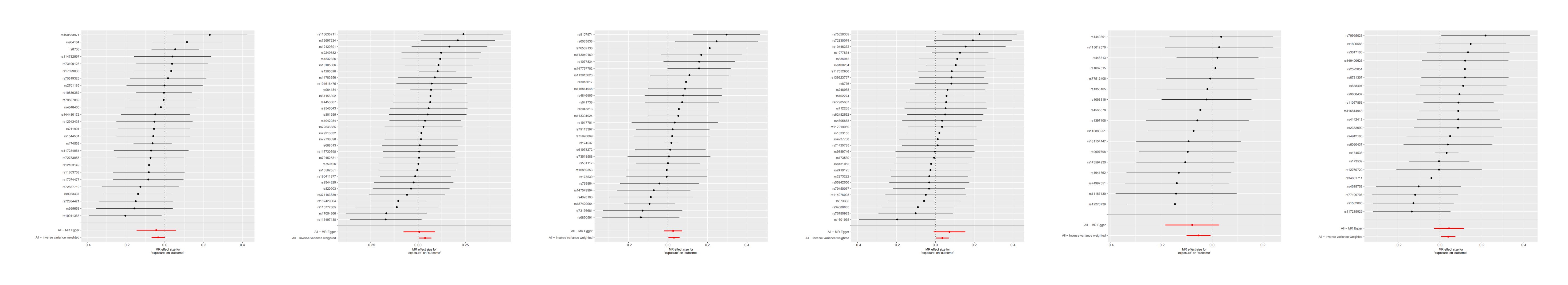

Supplement: Supplementary file 1 — Additional file 1: Figure S1. Forest plot. [file 13048_2025_1884_MOESM1_ESM.png]

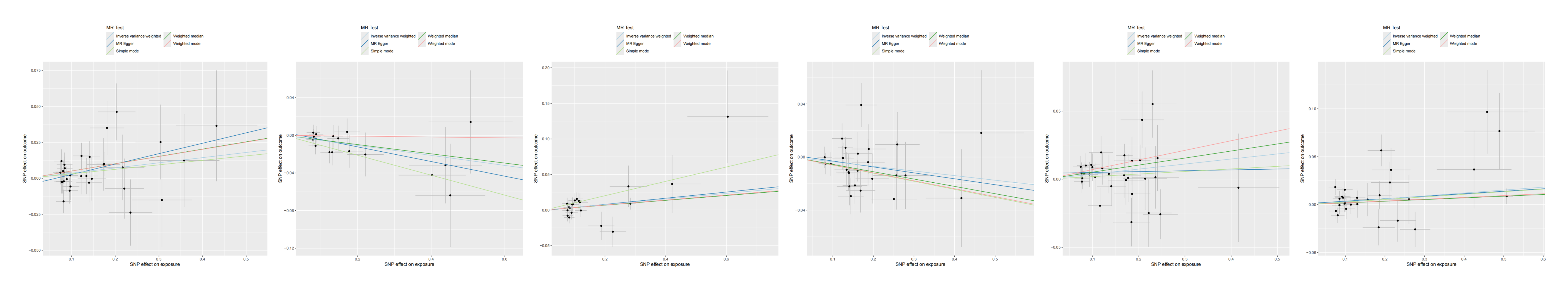

Supplement: Supplementary file 2 — Additional file 2: Figure S2. Scatter plot. [file 13048_2025_1884_MOESM2_ESM.png]

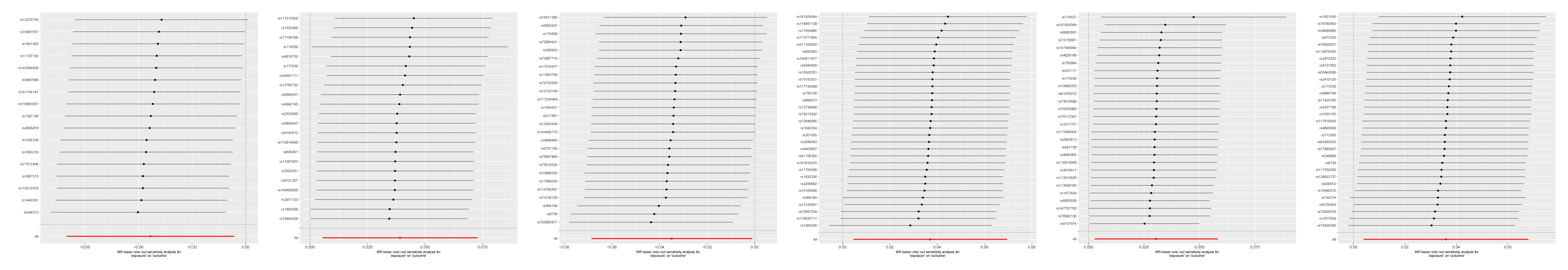

Supplement: Supplementary file 3 — Additional file 3: Figure S3. Sensitivity_analysis plot. [file 13048_2025_1884_MOESM3_ESM.png]

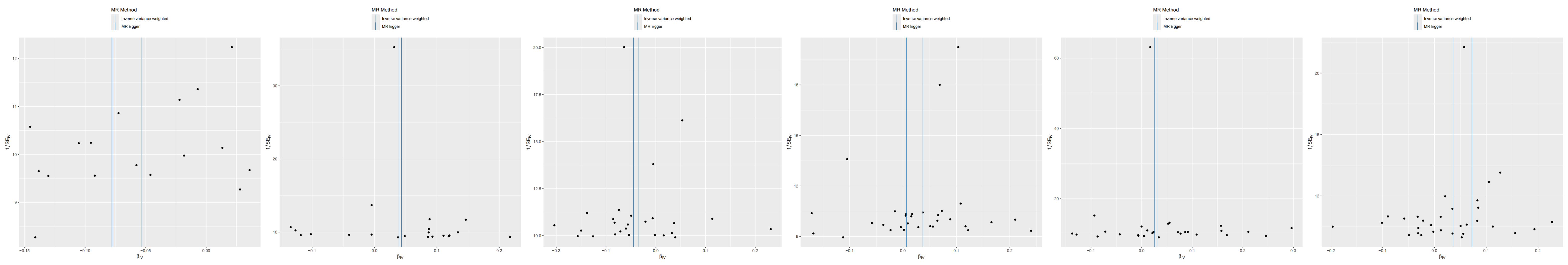

Supplement: Supplementary file 4 — Additional file 4: Figure S4. Funne lplo. [file 13048_2025_1884_MOESM4_ESM.png]
