# Supplementary material for: Identifying the mediating role of immune cells on the relationship between plasma lipidomes and PCOS: a two-step Mendelian randomization analysis
Source: J Ovarian Res. 2025 Dec 11;19:16. doi: 10.1186/s13048-025-01884-z (PMC12817645; doi:10.1186/s13048-025-01884-z)
Supplement: Supplementary file 5 — Additional file 5: Supplement table 1. BWMR results of plasma lipidomes on PCOS. [file 13048_2025_1884_MOESM5_ESM.docx]

**Supplement table 1. BWMR results of plasma lipidomes on PCOS.**

| ID | Method | Beta | lci95 | uci95 | OR | OR_lci95 | OR_uci95 | P value |
| --- | --- | --- | --- | --- | --- | --- | --- | --- |
| GCST90277313 | BWMR | 0.032433801 | 0.008435515 | 0.056432086 | 1.032965509 | 1.008471194 | 1.058054756 | 0.008074237 |
| GCST90277317 | BWMR | 0.03688542 | 0.003436396 | 0.070334443 | 1.037574129 | 1.003442308 | 1.072866934 | 0.030667445 |
| GCST90277326 | BWMR | -0.05397931 | -0.10286638 | -0.005092239 | 0.947451709 | 0.902247523 | 0.994920704 | 0.030452065 |
| GCST90277340 | BWMR | 0.041504852 | 0.0073181 | 0.075691605 | 1.04237822 | 1.007344943 | 1.078629879 | 0.017333208 |
| GCST90277366 | BWMR | -0.036464351 | -0.071777416 | -0.001151285 | 0.964192466 | 0.93073804 | 0.998849377 | 0.042980399 |
| GCST90277390 | BWMR | 0.039176595 | 0.005339928 | 0.073013261 | 1.039954118 | 1.00535421 | 1.075744803 | 0.023249052 |
